# Characterizing the Pathogenic, Genomic, and Chemical Traits of *Aspergillus fischeri*, a Close Relative of the Major Human Fungal Pathogen *Aspergillus fumigatus*

**DOI:** 10.1128/mSphere.00018-19

**Published:** 2019-02-20

**Authors:** Matthew E. Mead, Sonja L. Knowles, Huzefa A. Raja, Sarah R. Beattie, Caitlin H. Kowalski, Jacob L. Steenwyk, Lilian P. Silva, Jessica Chiaratto, Laure N. A. Ries, Gustavo H. Goldman, Robert A. Cramer, Nicholas H. Oberlies, Antonis Rokas

**Affiliations:** a Department of Biological Sciences, Vanderbilt University, Nashville, Tennessee, USA; b Department of Chemistry and Biochemistry, University of North Carolina at Greensboro, Greensboro, North Carolina, USA; c Department of Microbiology and Immunology, Geisel School of Medicine at Dartmouth, Hanover, New Hampshire, USA; d Faculdade de Ciencias Farmacêuticas de Ribeirão Preto, Universidade de São Paulo, São Paulo, Brazil; Carnegie Mellon University

**Keywords:** comparative genomics, evolution of virulence, fungal pathogenesis, *laeA*, secondary metabolism, specialized metabolism

## Abstract

Aspergillus fischeri is closely related to Aspergillus fumigatus, the major cause of invasive mold infections. Even though *A. fischeri* is commonly found in diverse environments, including hospitals, it rarely causes invasive disease. Why *A. fischeri* causes less human disease than A. fumigatus is unclear. A comparison of *A. fischeri* and A. fumigatus for pathogenic, genomic, and secondary metabolic traits revealed multiple differences in pathogenesis-related phenotypes. We observed that *A. fischeri* NRRL 181 is less virulent than A. fumigatus strain CEA10 in multiple animal models of disease, grows slower in low-oxygen environments, and is more sensitive to oxidative stress. Strikingly, the observed differences for some traits are of the same order of magnitude as those previously reported between A. fumigatus strains. In contrast, similar to what has previously been reported, the two species exhibit high genomic similarity; ∼90% of the A. fumigatus proteome is conserved in *A. fischeri*, including 48/49 genes known to be involved in A. fumigatus virulence. However, only 10/33 A. fumigatus biosynthetic gene clusters (BGCs) likely involved in secondary metabolite production are conserved in *A. fischeri* and only 13/48 *A. fischeri* BGCs are conserved in A. fumigatus. Detailed chemical characterization of *A. fischeri* cultures grown on multiple substrates identified multiple secondary metabolites, including two new compounds and one never before isolated as a natural product. Additionally, an *A. fischeri* deletion mutant of *laeA*, a master regulator of secondary metabolism, produced fewer secondary metabolites and in lower quantities, suggesting that regulation of secondary metabolism is at least partially conserved. These results suggest that the nonpathogenic *A. fischeri* possesses many of the genes important for A. fumigatus pathogenicity but is divergent with respect to its ability to thrive under host-relevant conditions and its secondary metabolism.

**IMPORTANCE** Aspergillus fumigatus is the primary cause of aspergillosis, a devastating ensemble of diseases associated with severe morbidity and mortality worldwide. A. fischeri is a close relative of A. fumigatus but is not generally observed to cause human disease. To gain insights into the underlying causes of this remarkable difference in pathogenicity, we compared two representative strains (one from each species) for a range of pathogenesis-relevant biological and chemical characteristics. We found that disease progression in multiple *A. fischeri* mouse models was slower and caused less mortality than A. fumigatus. Remarkably, the observed differences between *A. fischeri* and A. fumigatus strains examined here closely resembled those previously described for two commonly studied A. fumigatus strains, AF293 and CEA10. *A. fischeri* and A. fumigatus exhibited different growth profiles when placed in a range of stress-inducing conditions encountered during infection, such as low levels of oxygen and the presence of chemicals that induce the production of reactive oxygen species. We also found that the vast majority of A. fumigatus genes known to be involved in virulence are conserved in *A. fischeri*, whereas the two species differ significantly in their secondary metabolic pathways. These similarities and differences that we report here are the first step toward understanding the evolutionary origin of a major fungal pathogen.

## Abstract (precis)

Aspergillus fumigatus is the primary cause of aspergillosis, a devastating ensemble of diseases associated with severe morbidity and mortality worldwide. A. fischeri is a close relative of A. fumigatus but is not generally observed to cause human disease. To gain insights into the underlying causes of this remarkable difference in pathogenicity, we compared two representative strains (one from each species) for a range of pathogenesis-relevant biological and chemical characteristics. We found that disease progression in multiple *A. fischeri* mouse models was slower and caused less mortality than A. fumigatus. Remarkably, the observed differences between *A. fischeri* and A. fumigatus strains examined here closely resembled those previously described for two commonly studied A. fumigatus strains, AF293 and CEA10. *A. fischeri* and A. fumigatus exhibited different growth profiles when placed in a range of stress-inducing conditions encountered during infection, such as low levels of oxygen and the presence of chemicals that induce the production of reactive oxygen species. We also found that the vast majority of A. fumigatus genes known to be involved in virulence are conserved in *A. fischeri*, whereas the two species differ significantly in their secondary metabolic pathways. These similarities and differences that we report here are the first step toward understanding the evolutionary origin of a major fungal pathogen.

## INTRODUCTION

Aspergillosis is a major cause of human morbidity and mortality, resulting in over 200,000 life-threatening infections each year worldwide, and is primarily caused by the filamentous fungus Aspergillus fumigatus (1). Multiple virulence traits related to invasive aspergillosis (IA) are known for A. fumigatus, including growth at human body temperature and under low-oxygen conditions, the ability to acquire micronutrients such as iron and zinc in limiting environments, and the production of a diverse set of secondary metabolites (1).

Growth at human body temperature is a key trait for the survival of A. fumigatus inside mammalian hosts and may have arisen through adaptation to the warm temperatures present in decaying compost piles, one of the organism’s ecological niches (2–4). The primary route of A. fumigatus colonization and infection is through the lung, where oxygen levels have been observed to be as low as 2/3 of atmospheric pressure during infection. A successful response to this hypoxic environment is required for fungal pathogenesis (5, 6). Moreover, A. fumigatus produces a diverse set of small, bioactive molecules, known as secondary metabolites, which are biosynthesized in pathways that are often encoded by biosynthetic gene clusters (BGCs) (7). Some of these secondary metabolites and their regulators have been shown to contribute to disease in mouse models (8–10). Furthermore, a master regulator of secondary metabolism, *laeA*, is required for full virulence in IA mouse model studies (11, 12).

Species closely related to A. fumigatus are also capable of causing disease, but they are rarely observed in the clinic (1, 13–15). For example, Aspergillusfischeri is the closest evolutionary relative to A. fumigatus for which a genome has been sequenced (16, 17), but it is rarely reported to cause human disease (1). Recent evolutionary genomic analyses suggest that *A. fischeri* and A. fumigatus last shared a common ancestor approximately 4 million years ago (95% credible interval: 2 to 7 million years ago) (17). Why *A. fischeri*-mediated disease is less common than A. fumigatus-mediated disease remains an open question. Non-mutually exclusive possibilities include differences in ecological abundance, lack of species-level diagnosis of disease-causing strains in the clinic, and innate differences in pathogenicity and virulence between the two species.

Previous studies have suggested that the difference in the frequencies with which the two species cause disease is unlikely to be solely due to ecological factors, as both can be isolated from a variety of locales, including soils, fruits, and hospitals (18–20). For example, approximately 2% of the fungi isolated from the respiratory intensive care unit at Beijing Hospital were *A. fischeri* compared to approximately 23% of fungal species identified as A. fumigatus (20). While *A. fischeri* is easily isolated from a variety of environments, only a few cases of human infections have been reported (21–24). Furthermore, numerous recent epidemiological studies from multiple countries that used state-of-the-art molecular typing methods were able to identify several rarely isolated pathogenic species closely related to A. fumigatus, such as Aspergilluslentulus and Aspergillusudagawae, as the source of 10 to 15% of human infections but did not identify *A. fischeri* in any patient sample (13, 14, 25–27).

If ecological factors and lack of precision in species identification do not convincingly explain why *A. fischeri* is nonpathogenic and A. fumigatus is pathogenic, other factors must be responsible. An initial genomic comparison between strains of A. fumigatus, *A. fischeri*, and the more distantly related Aspergillus clavatus identified 818 genes that were A. fumigatus specific (16). These genes were enriched for functions associated with carbohydrate transport and catabolism, secondary metabolite biosynthesis, and detoxification (16), raising the possibility that the observed differences in pathogenicity observed between *A. fischeri* and A. fumigatus have a molecular basis.

To gain further insight into why *A. fischeri*-mediated disease is less abundant than A. fumigatus-mediated disease, we took a multipronged approach to investigate phenotypic, genomic, and chemical differences between *A. fischeri* strain NRRL 181 and A. fumigatus strain CEA10. We observed that while *A. fischeri* is able to cause fatal disease in multiple animal models, its disease progression and response to multiple host-relevant stresses are markedly reduced compared to A. fumigatus CEA10. We also found that while the two organisms’ genomes are in general very similar, the sets of secondary metabolite pathways in each of them exhibit a surprisingly low level of overlap. Examination of the secondary metabolite profile of *A. fischeri* identified both previously isolated and novel compounds. Finally, construction of a mutant *A. fischeri* strain that lacked the *laeA* gene, a master regulator of secondary metabolism, and examination of its chemical profile suggested that LaeA-mediated regulation of secondary metabolism in *A. fischeri* closely resembles that of A. fumigatus. These results begin to reveal the molecular differences between *A. fischeri* and A. fumigatus related to fungal pathogenesis and suggest that a functional evolutionary genomic comparison between pathogenic and nonpathogenic species closely related to A. fumigatus harbors great promise for generating insights into the evolution of fungal disease.

## RESULTS

### *A. fischeri* is less virulent than A. fumigatus in multiple animal models of invasive pulmonary aspergillosis (IPA).

**FIG 1:**
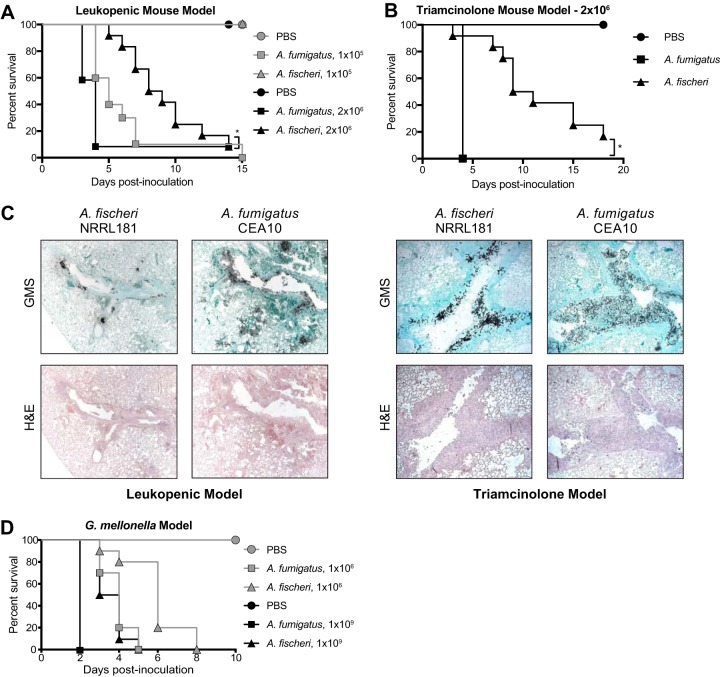
*A. fischeri* is significantly less virulent than A. fumigatus in multiple murine models of invasive pulmonary aspergillosis. (A) Cumulative survival of mice inoculated with 1 × 10^5^ (gray) or 2 × 10^6^ (black) conidia in a leukopenic model of IPA. For the inoculations of 1 × 10^5^ conidia, 10 mice were infected per group, and for the inoculations of 2 × 10^6^ conidia, 12 mice were infected per group. For each PBS control, 4 mice were inoculated. *, *P* = 0.0098 by log rank test and *P* = 0.0002 by Gehan-Breslow-Wilcoxon test. (B) Cumulative survival of mice inoculated with 2 × 10^6^ conidia in a steroid model of IPA. *n* = 12/group, 4 for PBS. *, *P* = <0.0001 by log rank and Gehan-Breslow-Wilcoxon tests. (C) Histological sections from 3 days postinoculation in a leukopenic (left) and steroid (right) model of IPA, stained with H&E and GMS. Images were acquired at 100×. (D) Cumulative survival of G. mellonella larvae inoculated with 1 × 10^6^ (gray) or 1 × 10^9^ (black) conidia. Ten larvae were used per condition in all assays. Survival curves for *A. fischeri* and A. fumigatus were significantly different (*P* < 0.003) in both log rank and Gehan-Breslow-Wilcoxon tests for both inocula.

In contrast to A. fumigatus-mediated disease, only a few cases of invasive fungal infections have been reported to be caused by *A. fischeri* (21–24). Given this contrast, we utilized two immunologically distinct murine IPA models to assess differences in pathogenicity and virulence between the two species. In a leukopenic murine model, *A. fischeri* NRRL 181 was significantly less virulent than A. fumigatus CEA10, in a dose-dependent manner (Fig. 1A). Using an inoculum of 1 × 10^5^ conidia, *A. fischeri* was completely attenuated in virulence, with 100% murine survival by day 15 post-fungal challenge. In contrast, inoculation with A. fumigatus resulted in 100% murine mortality by day 15 (Fig. 1A). Using a higher dose (2 × 10^6^) of conidia, both strains caused 90% mortality by day 14; however, the disease progression was markedly different. Eighty percent of mice inoculated with A. fumigatus succumbed to infection by day 4, whereas in mice inoculated with *A. fischeri*, mortality started occurring on day 5, and then one or two mice succumbed each day until day 14 (Fig. 1A). Thus, despite the similar overall mortality at higher fungal challenge doses, *A. fischeri* is substantially less virulent than A. fumigatus in a leukopenic murine IPA model.

To better understand what is happening *in vivo* during disease progression with *A. fischeri* NRRL 181 and A. fumigatus CEA10, we performed histological analyses on lungs from the leukopenic model 3 days postinoculation. Histological sections were stained with Gomori methenamine silver (GMS) to visualize fungal burden and with hematoxylin and eosin (H&E) stain to visualize host-related pathology (Fig. 1C). Leukopenic mice inoculated with A. fumigatus had an overall greater number of fungal lesions per animal than mice inoculated with *A. fischeri*. These A. fumigatus lesions contained a greater amount of fungus, which coincided with increased tissue necrosis surrounding the lesions. There was also a greater number of A. fumigatus lesions with fungi invading from the larger airways into the lung parenchyma. Meanwhile, the *A. fischeri* lesions were largely maintained within the larger airways. A striking difference between the lesions caused by the two species was in the appearance of the hyphae of the two species, where *A. fischeri* filaments were much shorter than those of A. fumigatus. We hypothesize that in the leukopenic mouse, *A. fischeri* is unable to grow as well as A. fumigatus, resulting in smaller and fewer fungal lesions, shorter fungal filaments, and attenuation of virulence.

As the patient population at risk for IA continues to change and grow (28), we next tested a nonleukopenic, triamcinolone (steroid)-induced immune suppression model and observed a significant reduction in virulence of *A. fischeri* compared to A. fumigatus (*P* < 0.0001 by log rank and Gehan-Breslow-Wilcoxon tests). All mice inoculated with A. fumigatus succumbed to infection by day 4; however, similar to the leukopenic model, mice inoculated with *A. fischeri* had significantly slower disease progression as monitored by Kaplan-Meier analyses (Fig. 1B).

Histological analyses were also carried out on lungs from the steroid model 3 days post-fungal inoculation (Fig. 1C). Overall, mice inoculated with *A. fischeri* in the steroid model had similar numbers of fungal lesions as those inoculated with A. fumigatus, but the lesions caused by the two species were phenotypically distinct (Fig. 1C). In larger terminal bronchioles infected with A. fumigatus, there was greater fungal growth per lesion, and the growth was observed throughout the bronchiole itself, extending well into the lumen. These lesions were accompanied by substantial granulocytic inflammation and obstructed the airways surrounding the hyphae (Fig. 1C). In the lesions containing *A. fischeri*, the fungal growth was contained to the epithelial lining of the bronchioles. This pattern of growth was accompanied by inflammation at the airway epithelia, leaving the airway lumen largely unobstructed (Fig. 1C). The lack of airway obstruction during *A. fischeri* infection and reduced inflammation may contribute to the reduced virulence compared to A. fumigatus in this murine model.

Although the distribution of the fungal lesions varied, there was still significant fungal growth in the steroid immunosuppressed mice infected with *A. fischeri*, suggesting that *A. fischeri* is capable of growing within the immunocompromised murine host. Indeed, we tested the growth rate of *A. fischeri* and A. fumigatus in lung homogenate as a proxy for growth capability within the nutrient environment of the host and observed no difference between the two strains (see Fig. S1 in the supplemental material). These experiments show that in multiple models of fungal disease, *A. fischeri* is less virulent than A. fumigatus even though it is able to grow within the immunocompromised murine lung.

10.1128/mSphere.00018-19.1 FIG S1 A. fumigatus grows slower than *A. fischeri* in glucose minimal medium (GMM) but at the same speed as *A. fischeri* in lung homogenate medium. A. fumigatus CEA10 or *A. fischeri* NRRL 181 was cultured in flat-bottom 96-well plates at 2 × 10^4^ conidia per well. Conidia were added in 20 µl of 0.01% Tween 80, and medium was carefully pipetted over the inoculum into each well. Lung homogenate was generated according to reference 29. Plates were incubated for 7 hours at 37°C before measurements at 405 nm were taken every 10 min. Mean and SEM for eight technical replicates; data are representative of three biological replicates. Download FIG S1, PDF file, 0.2 MB. Copyright © 2019 Mead et al. 2019 Mead et al. This content is distributed under the terms of the Creative Commons Attribution 4.0 International license.

We observed similar pathogenicity and virulence results when using the Galleria mellonella insect larva model of aspergillosis (Fig. 1D). Both low (1 × 10^6^ conidia)- and high (1 × 10^9^ conidia)-inoculum experiments showed significant differences between the disease progression of *A. fischeri* (slower) and A. fumigatus (faster) in this insect model of fungal pathogenicity.

### Compared to A. fumigatus, *A. fischeri* differs in its response to several host-relevant stresses.

**FIG 2:**
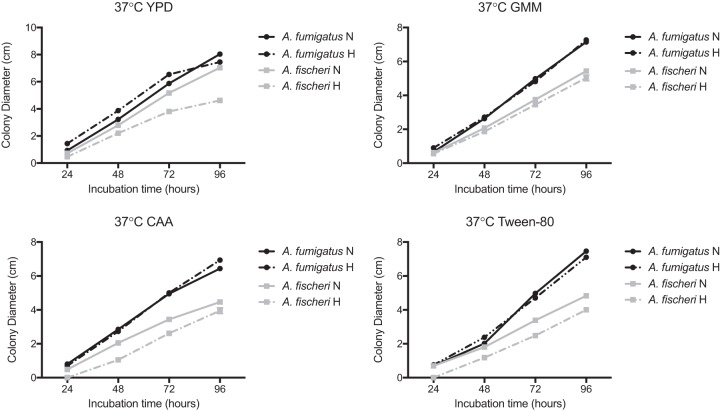
*A. fischeri* is unable to thrive under suboptimal metabolic conditions at 37°C. A total of 1 × 10^3^ conidia were point inoculated on each plate, and then plates were incubated at 37°C in normoxia (N; ∼21% oxygen, 5% CO_2_) or hypoxia (H; 0.2% O_2_, 5% CO_2_); colony diameter was measured every 24 h. Mean and SEM for triplicates. CAA, Casamino Acids; GMM, glucose minimal medium.

**FIG 3:**
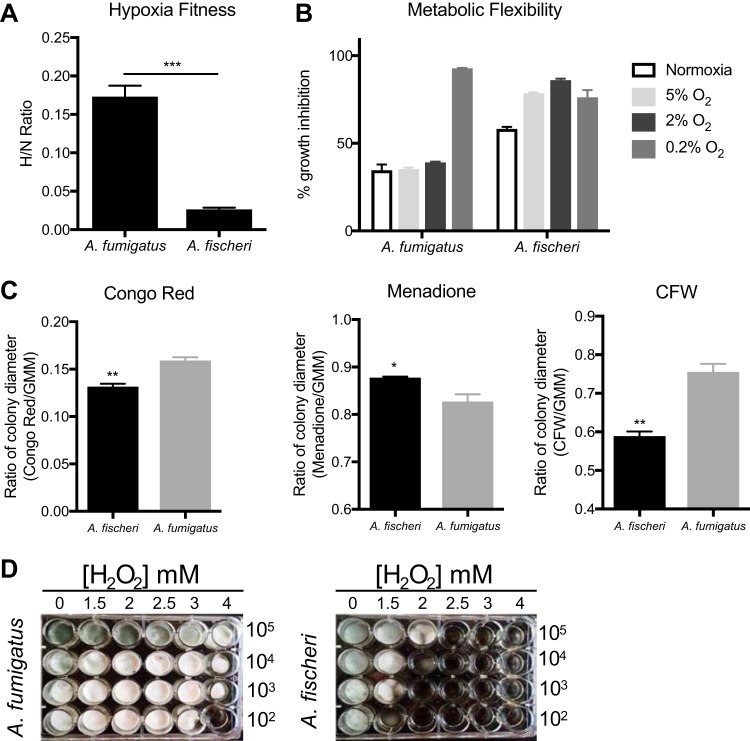
*A. fischeri* is more susceptible to multiple host-relevant stresses than A. fumigatus. (A) Fitness ratio of A. fumigatus or *A. fischeri* during hypoxic versus normoxic growth (measured as the dry weight of cultures). Data represent mean and SEM for biological triplicates; ***, *P* = 0.0006 by Student’s *t* test. (B) Growth inhibition of strains grown on 1% lactate minimal medium with 0.1% 2-deoxyglucose (2-DG) under a range of low-oxygen conditions. (C) A. fumigatus and *A. fischeri* were grown in the presence of the cell wall-perturbing agent Congo red (0.5 mg/ml), the oxidative stressor menadione (20 µM), or the chitin-perturbing agent calcofluor white (CFW; 25 µg/ml). Plates were grown for 96 h at 37°C and 5% CO_2_. For all plates except Congo red and its GMM control, 1 × 10^3^ spores were plated. For Congo red and the control GMM plate, 1 × 10^5^ spores were plated. Student’s *t* test was performed, and significance was as follows: *, *P* < 0.05; **, *P* < 0.01. (D) Strains were grown for 48 h at 37°C in liquid complete medium supplemented with increasing concentrations of hydrogen peroxide.

Our *in vivo* experiments suggested that the lower virulence of *A. fischeri* is not the result of an inability to grow within the host *per se*. Therefore, we hypothesized that *A. fischeri* is unable to mitigate stresses encountered in the host as effectively as its close evolutionary relative A. fumigatus. Nutrient fluctuation is a stress encountered *in vivo* during A. fumigatus infection (29), and to assess differences in metabolic plasticity between the two species, we measured the two organisms’ growth on media supplemented with glucose, fatty acids (Tween 80), or Casamino Acids. Because low oxygen tension is a significant stress encountered during infection (5), and recently, fitness in low oxygen has been correlated with virulence of A. fumigatus (30), we measured growth of both species at 37°C under both normoxic (ambient air) and hypoxia-inducing (0.2% O_2_, 5% CO_2_) conditions. In normoxia with glucose, fatty acids (Tween 80), or Casamino Acids supplied as the carbon source, radial growth of *A. fischeri* was reduced compared to A. fumigatus (Fig. 2). However, on rich media both organisms grew equally well (Fig. 2). We also observed a lower growth rate of *A. fischeri* than A. fumigatus in the first 16 h of culture in liquid media supplied with glucose at 37°C. At 30°C, *A. fischeri* grew the same as, or better than, A. fumigatus except on Tween 80, where A. fumigatus had a slight advantage (Fig. S2). Also, *A. fischeri* grew substantially worse than A. fumigatus at 44°C (Fig. S2), a temperature rarely observed in the patient but easily observed in compost piles. To determine relative fitness in hypoxic liquid environments, we measured the ratio of biomass in liquid culture in ambient air (normoxia) versus hypoxic (0.2% O_2_, 5% CO_2_) conditions. *A. fischeri* showed significantly lower fitness under hypoxic conditions, with about an 8.5-fold-lower biomass than A. fumigatus (Fig. 3A). These data suggest that *A. fischeri* is less fit than A. fumigatus at 37°C and under low-oxygen conditions, both of which have been shown to impact fungal virulence.

10.1128/mSphere.00018-19.2 FIG S2 *A. fischeri* and A. fumigatus exhibit similar growth patterns at 30°C but not at 44°C. (A to D) Conidia at 1 × 10^3^ were point inoculated on each plate, and plates were then incubated at 30°C in normoxia (∼21% oxygen, 5% CO_2_); colony diameter was measured every 24 hours. Mean and SEM for triplicates. Tween 80, 1% Tween 80 provided as sole carbon source; CAA, Casamino Acids; GMM, glucose minimal medium. (E) Error bars indicate standard deviations between biological duplicates (**, *P* value < 0.005 in a paired, equal-variance Student *t* test). Download FIG S2, PDF file, 0.7 MB. Copyright © 2019 Mead et al. 2019 Mead et al. This content is distributed under the terms of the Creative Commons Attribution 4.0 International license.

Metabolic flexibility, or the ability for an organism to utilize multiple carbon sources simultaneously, has been suggested to provide a fitness advantage to Candida albicans during *in vivo* growth (31). Metabolic flexibility can be characterized using the glucose analog 2-deoxyglucose (2-DG), in combination with an alternative carbon source available *in vivo*, such as lactate. 2-DG triggers carbon catabolite repression, which shuts down alternative carbon utilization pathways. However, in C. albicans this shutdown is delayed and growth occurs on lactate with 2-DG (31, 32). We tested the metabolic flexibility of both A. fumigatus and *A. fischeri* and observed that while both species can grow in the presence of 2-DG on lactate, the growth inhibition of *A. fischeri* is higher (∼60%) than that of A. fumigatus (∼35%; Fig. 3B). Even under low-oxygen conditions (5% and 2%), A. fumigatus maintains this metabolic flexibility except under extremely low oxygen conditions (0.2%), whereas *A. fischeri* shows even greater inhibition at all oxygen tensions of 5% or below. These data suggest that while both species exhibit some level of metabolic flexibility, A. fumigatus appears more metabolically flexible under a wider range of conditions than *A. fischeri*.

Next, we measured the susceptibility of *A. fischeri* to oxidative stress, cell wall stress, and antifungal drugs as they are stresses that are encountered during infection and treatment (33). Interestingly, we observed that *A. fischeri* is more resistant to the intracellular oxidative stress agent menadione than A. fumigatus but more susceptible to the external oxidative stress agent hydrogen peroxide (Fig. 3C and D). As the *in vivo* levels of inflammation caused by the two species appeared different, we indirectly tested for differences in cell wall pathogen-associated molecular patterns using the cell wall-perturbing agents Congo red and calcofluor white. A. fumigatus was significantly more resistant to both agents than *A. fischeri* (Fig. 3C), suggesting differences in the response to cell wall stress or in the composition and organization of the cell wall between the two species. These differences are likely important for host immune cell recognition and interaction, which in turn influence pathology and disease outcome.

Last, *A. fischeri* showed enhanced resistance relative to A. fumigatus for three of the four antifungal drugs tested (Table 1), consistent with previous experiments (34). Overall, our phenotypic data show that the response of *A. fischeri* to host-related stresses and antifungals is substantially different from that of A. fumigatus. Furthermore, our results suggest that increased growth capability of A. fumigatus in low oxygen and in high temperatures are two important attributes that likely contribute to its pathogenic potential compared to *A. fischeri*.

**TABLE 1:** *A. fischeri* shows enhanced resistance relative to *A. fumigatus* for several antifungal drugs

Strain	MIC of drug (µg/ml)			MEC of caspofungin (µg/ml)
	Posaconazole	Voriconazole	Itraconazole	
*A. fumigatus*	0.7	0.8	5	0.09
*A. fischeri*	2.4	>4	>24	0.06

### The proteomes of A. fumigatus and *A. fischeri* are highly similar, but their secondary metabolic pathways show substantial divergence.

The large differences in virulence and virulence-related traits we observed between A. fumigatus and *A. fischeri* led us to investigate the genotypic differences that could be responsible. To describe the genomic similarities and differences between A. fumigatus and *A. fischeri*, we determined how many orthologous proteins and how many species-specific proteins were present in each genome using a reciprocal best BLAST hit approach (35). We identified 8,737 proteins as being shared between the two species (Fig. S3), representing 88% and 84% of the A. fumigatus and *A. fischeri* proteomes, respectively, and 1,684 *A. fischeri*-specific proteins (16% of its proteome) and 1,189 A. fumigatus-specific proteins (12% of its proteome). These results are similar to what had previously been reported in an early analysis of the *A. fischeri* genome (16).

10.1128/mSphere.00018-19.3 FIG S3 The genomes of A. fumigatus and *A. fischeri* are largely similar, but their secondary metabolic pathways are quite divergent. Left, Venn diagram showing the sets of *A. fischeri*-specific proteins, shared orthologous proteins, and A. fumigatus-specific proteins encoded in each genome. Numbers below each species name indicate the total number of proteins encoded in that genome. Right, Venn diagram showing the sets of *A. fischeri*-specific BGC proteins, shared BGC proteins, and A. fumigatus-specific BGC proteins. Numbers below each species name indicate the total number of BGC proteins encoded in that genome. In each diagram, circles are proportional to the number of proteins they contain. Download FIG S3, PDF file, 0.1 MB. Copyright © 2019 Mead et al. 2019 Mead et al. This content is distributed under the terms of the Creative Commons Attribution 4.0 International license.

To narrow our search for genes that are absent in *A. fischeri* but are important for A. fumigatus disease, we compiled a list of 49 A. fumigatus genes considered to be involved in virulence (see Table S1 in the Figshare document at https://doi.org/10.6084/m9.figshare.7149167) based on two previously published articles (36, 37) and extensive literature searches of our own. We observed that all but one of these virulence-associated genes were also present in *A. fischeri*, a surprising finding considering the substantial differences observed between the two species in our animal models of infection. The virulence-associated gene not present in *A. fischeri* is *pesL* (Afu6g12050), a nonribosomal peptide synthase that is essential for the synthesis of the secondary metabolite fumigaclavine C and required for virulence in the *Galleria* model of A. fumigatus infection (38).

Since the only previously described A. fumigatus virulence-associated gene not present in the *A. fischeri* genome (i.e., *pesL*) is involved in secondary metabolism and a previous study suggested that secondary metabolism is not conserved between these two species (39), we investigated the differences between the repertoire of secondary metabolic pathways present in A. fumigatus and *A. fischeri*. Using the program antiSMASH (40), we identified 598 genes distributed among 33 BGCs in A. fumigatus (see Table S2 in the Figshare document at https://doi.org/10.6084/m9.figshare.7149167) and 786 genes spread out over 48 BGCs in *A. fischeri* (see Table S3 in the Figshare document at https://doi.org/10.6084/m9.figshare.7149167). Of these 598 A. fumigatus genes, 407 (68%) had an orthologous gene that was part of an *A. fischeri* BGC. This level of conservation of BGC genes (68%) is much lower than the amount of conservation observed for the rest of the proteome (88%), illustrating the high rate at which fungal metabolic pathways evolve (41, 42).

**FIG 4:**
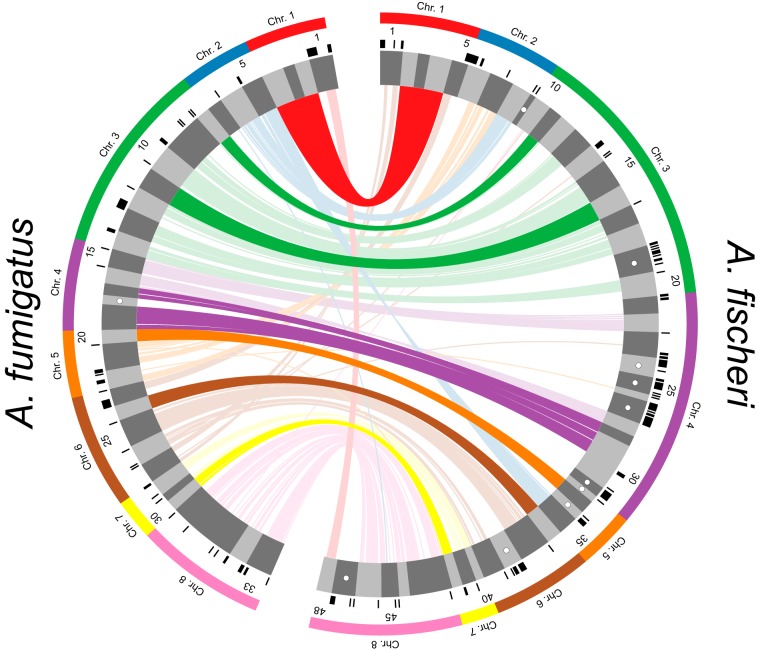
Biosynthetic gene clusters (BGCs) of A. fumigatus and *A. fischeri* show substantial evolutionary divergence. Predicted BGCs are shown in the inner track and are alternatively colored dark and light gray, and their size is proportional to the number of genes in them. Black ticks on the exterior of the cluster track indicate genes that possess an ortholog in the other species but are not in a BGC in the second species. White dots indicate species-specific clusters. Solid bars on the exterior correspond to the chromosome on which the BGCs below them reside. Genes are connected to their orthologs in the other species with dark lines if >90% of the BGC genes in A. fumigatus are conserved in the same BGC in *A. fischeri*. Lighter lines connect all other orthologs that are present in both species’ sets of BGCs. The image was made using Circos version 0.69-4 (86).

We next directly compared the BGCs of the two organisms. An A. fumigatus BGC was considered conserved in *A. fischeri* if ≥90% of its genes were also present in an *A. fischeri* BGC and vice versa. We found that only 10/33 A. fumigatus BGCs are conserved in *A. fischeri* and only 13/48 *A. fischeri* BGCs are conserved in A. fumigatus (Fig. 4), a finding consistent with previous results (39) and the low conservation of individual secondary metabolic genes between the two species. While only 10 A. fumigatus BGCs were conserved in *A. fischeri*, many other BGCs contained one or more orthologs of genes in *A. fischeri* BGCs.

Only one BGC (cluster 18) was completely A. fumigatus specific. Interestingly, our previous examination of the genomes of 66 A. fumigatus strains showed that this BGC was a “jumping cluster,” as it was found to be present in only 5 strains and to reside in three distinct genomic locations (42). Conversely, there are 10 *A. fischeri*-specific BGCs that do not have orthologs in BGCs in A. fumigatus. One of these BGCs is responsible for making helvolic acid (a gene cluster known to be absent from the *A. fumigatus* strain CEA10 but present in strain Af293 [42]), but the other 9 have not been biochemically connected to any metabolite.

All the genes required for the production of the mycotoxin gliotoxin are located in a BGC in *A. fischeri* (Fig. S4) and are in fact similar to their A. fumigatus orthologs (43), even though *A. fischeri* is not known to produce this mycotoxin (44). Both the gliotoxin and acetylaszonalenin BGCs are adjacent to one another in the *A. fischeri* genome (Fig. S4). In A. fumigatus, the gliotoxin BGC is immediately next to what appears to be a truncated version of the acetylaszonalenin BGC that lacks portions of the nonribosomal peptide synthase and acetyltransferase genes as well as the entire indole prenyltransferase gene required for acetylaszonalenin production. The close proximity of these two BGCs is noteworthy, as it is similar to previously reported “superclusters” in A. fumigatus and A. fumigatus-related strains (45). These superclusters have been hypothesized to be “evolutionary laboratories” that may give rise to new compounds and pathways (42).

10.1128/mSphere.00018-19.4 FIG S4 The acetylaszonalenin and gliotoxin biosynthetic gene clusters in A. fumigatus and *A. fischeri* are located immediately next to one another. The portions of clusters 37 and 25 from *A. fischeri* and A. fumigatus, respectively, that are known to contain the previously characterized acetylaszonalenin (96) and gliotoxin (43) BGCs are shown. Genes colored in shades of green are involved in the acetylaszonalenin biosynthetic pathway. Dark green, *anaPS* (nonribosomal peptide synthase); light green, *anaAT* (acetyltransferase); green, *anaPT* (prenyltransferase); orange, gliotoxin biosynthetic genes. Gray arrow, syntenic gene in both species not involved in gliotoxin synthesis. Sequences that are similar to one another (based on blastn scores) are marked by gray parallelograms. Image was made using Easyfig version 2.2.2 (88). Download FIG S4, PDF file, 0.1 MB. Copyright © 2019 Mead et al. 2019 Mead et al. This content is distributed under the terms of the Creative Commons Attribution 4.0 International license.

### Isolation and characterization of three new compounds from *A. fischeri*.

The relatively low level of conservation of BGCs we observed between A. fumigatus and *A. fischeri* led us to characterize the secondary metabolites produced by *A. fischeri* (Fig. S5) (46–50). The one strain-many compounds (OSMAC) approach was used to alter the secondary metabolites being biosynthesized in order to produce a diverse set of molecules (51–54). Depending on the medium on which it was grown, *A. fischeri* produced as few as 4 (yeast extract soy peptone dextrose agar [YESD]) or as many as 10 (oatmeal agar [OMA]) compounds (Fig. S6). These results showed that culture medium influences the biosynthesis of secondary metabolites in *A. fischeri*, a phenomenon observed in many other fungi (52, 55).

10.1128/mSphere.00018-19.5 FIG S5 A custom chemical analysis protocol was developed for studying the metabolites produced by *A. fischeri*. Approximately 60 ml of 1:1 CH_3_OH-CH_3_Cl was added to cultures of *Aspergillus fischeri* grown on solid-state fermentation for 2 weeks. The cultures were then chopped thoroughly with a large scalpel and shaken for 16 hours using an orbital shaker. The liquid culture was then vacuum filtered and concentrated using 90 ml CH_3_Cl and 150 ml water and transferred into a separatory funnel. The organic (bottom) layer was drawn off and evaporated to dryness. The dried, desugared extract was reconstituted in 100 ml of 1:1 CH_3_OH-CH_3_CN and 100 ml of hexane. The biphasic solution was shaken vigorously and transferred to a separatory funnel. The CH_3_OH-CH_3_CN layer was evaporated to dryness under vacuum, producing a defatted extract. The extract was then subdivided into several peaks or fractions using flash chromatography. The subfractions were further separated using HPLC until pure compounds were isolated. The pure compounds were subjected to UPLC-MS analysis to establish the molecular formula and fragmentation patterns. Finally, pure compounds were identified using both NMR analysis and information from UPLC-MS data. Download FIG S5, PDF file, 0.9 MB. Copyright © 2019 Mead et al. 2019 Mead et al. This content is distributed under the terms of the Creative Commons Attribution 4.0 International license.

10.1128/mSphere.00018-19.6 FIG S6 *A. fischeri* produces different numbers of metabolites, depending on the medium it is grown on. Base peak chromatograms as measured by LC-MS, illustrating how the chemistry profiles varied based on growth conditions. PDA + ab was used as the chemical control to observe the differences in the secondary metabolites, due to it being the medium in which *A. fischeri* is stored. There were overall no chemical differences observed between the different variations of PDA medium. Each peak (which indicates different chemical entities) was observed in the three PDA variations, albeit at fluctuating intensities. SDA, PYG, and YESD produced the majority of the peaks observed in PDA, but it also lacked some observed peaks, indicating that these growth conditions were not chemically favored. CYA produced the majority of the peaks, as well as an additional peak that was observed at a much lower intensity in PDA. However, this peak was similarly observed in OMA. OMA produced similar peaks as those observed in PDA, but with higher intensity. Due to this, OMA was selected for further study. The gray boxes indicate differences in the observed peaks from PDA. See the Figshare document at https://doi.org/10.6084/m9.figshare.7149167 for more information. Download FIG S6, PDF file, 0.6 MB. Copyright © 2019 Mead et al. 2019 Mead et al. This content is distributed under the terms of the Creative Commons Attribution 4.0 International license.

**FIG 5:**
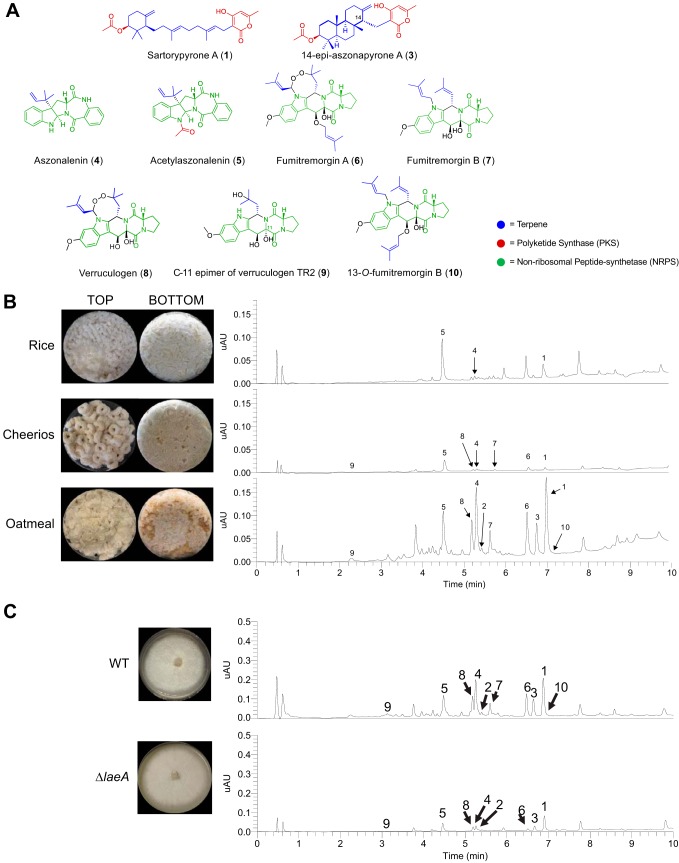
Secondary metabolite production in *A. fischeri*. (A) Compounds isolated from *A. fischeri*: 1, sartorypyrone A; 2, sartorypyrone E; 3, 14-epimer aszonapyrone A; 4, aszonalenin; 5, acetylaszonalenin; 6, fumitremorgin A; 7, fumitremorgin B; 8, verruculogen; 9, C-11 epimer verruculogen TR2; and 10, 13-*O*-prenyl-fumitremorgin B. The color coding indicates which putative class the molecule belongs to, e.g., terpenes, PKS, or NRPS. (B) (Top) *Aspergillus fischeri* was initially grown on rice for 2 weeks and then extracted using methods outlined in Fig. S5. The rice culture yielded compounds 1, 4, and 5. (Middle) *A. fischeri* was grown on multigrain Cheerios for 2 weeks, which yielded compounds 1 and 4 to 9. (Bottom) *A. fischeri* grown on Quaker oatmeal for 2 weeks. All compounds that were previously isolated in rice and multigrain Cheerios cultures in addition to three new compounds (2, 3, and 10) were found in the oatmeal culture. All pictures depict fungi growing in 250-ml Erlenmeyer flasks; the left panel indicates the top view, while the right panel shows the bottom view. All chromatographic profiles have been normalized to the highest μAU value. (C) *A. fischeri* wild-type and Δ*laeA* strains were grown on solid breakfast oatmeal for 2 weeks and extracted using organic solvents as indicated previously. The crude desugared and defatted extracts were run using UPLC-MS at a concentration of 2 mg/ml with 5 μl being injected for analysis. The chromatographic profiles were normalized to the highest μAU value. Mass spectrometry analysis indicated the presence of secondary metabolites 1 to 10 within the wild type, and only compounds 1 to 6, 8, and 9 were seen in the *ΔlaeA* mutant. All pictures show *A. fischeri* grown on oatmeal agar in petri plates.

To characterize the peaks of interest we observed when *A. fischeri* was grown on OMA, we increased the size of our fungal cultures; doing so yielded seven previously isolated compounds (sartorypyrone A [compound 1], aszonalenin [compound 4], acetylaszonalenin [compound 5], fumitremorgin A [compound 6], fumitremorgin B [compound 7], verruculogen [compound 8], and the C-11 epimer of verruculogen TR2 [compound 9]) and three newly biosynthesized secondary metabolites (sartorypyrone E [compound 2], 14-epiaszonapyrone A [compound 3], and 13-*O*-fumitremorgin B [compound 10]). Two of the secondary metabolites were new compounds (compounds 2 and 3), and one was a new natural product (compound 10) (Fig. 5B). The structures for all 10 compounds were determined using a set of spectroscopic (1D and 2D NMR) and spectrometric techniques (high-resolution mass spectrometry [HRMS]). Our data for sartorypyrone A (compound 1) (56), aszonalenin (compound 4) (57, 58), acetylaszonalenin (compound 5) (56, 59), fumitremorgin A (compound 6) (60, 61), fumitremorgin B (compound 7) (62–64), verruculogen (compound 8) (65, 66), and the C-11 epimer of verruculogen TR2 (compound 9) (66) correlated well with literature values. The structures of 14-epiaszonapyrone A (compound 3) and 13-*O*-prenyl fumitremorgin B (compound 10) were fully characterized in this study (see Figshare document at https://doi.org/10.6084/m9.figshare.7149167); the structure elucidation of sartorypyrone E (compound 2) is ongoing and will be reported in detail in a forthcoming manuscript.

Since four secondary metabolites (compounds 5 to 8) from *A. fischeri* had also been reported from A. fumigatus (Table 2), we hypothesized that the mechanisms *A. fischeri* employs to regulate its secondary metabolism would also be similar to those used by A. fumigatus. To test this hypothesis, we constructed a deletion mutant of *laeA* in *A. fischeri* (Fig. S7). LaeA is a master regulator of secondary metabolism in A. fumigatus and a variety of other fungi (67–69). Both the wild-type and Δ*laeA* strains of *A. fischeri* were subjected to LC-MS analysis. The chromatographic profile of the Δ*laeA* strain showed mass data that corresponded to sartorypyrone A (compound 1), sartorypyrone E (compound 2), 14-epiaszonapyrone A (compound 3), aszonalenin (compound 4), acetylaszonalenin (compound 5), fumitremorgin A (compound 6), verruculogen (compound 8), and the C-11 epimer of verruculogen TR2 (compound 9). However, the relative abundance of compounds present was very low compared to the wild type (Fig. 5C). Fumitremorgin B (compound 7) and 13-*O*-prenyl-fumitremorgin B (compound 10) were not produced by the Δ*laeA* mutant at all.

**TABLE 2:** Secondary metabolites isolated from *A. fischeri* that have been reported from *A. fumigatus*

Secondary metabolite (compound no.)	Presence in strain:	
	*A. fischeri*	*A. fumigatus*
Sartorypyrone A (1)	+	−
Sartorypyrone E (2)	+	−
14-Epiaszonapyrone A (3)	+	−
Aszonalenin (4)	+	−
Acetylaszonalenin (5)	+	+
Fumitremorgin A (6)	+	+
Fumitremorgin B (7)	+	+
Verruculogen (8)	+	+
C-11 epimer of verruculogen TR2 (9)	+	−
13-*O*-Fumitremorgin B (10)	+	−

10.1128/mSphere.00018-19.7 FIG S7 Southern blotting confirms construction of the Δ*laeA* mutant. A 1-kb probe recognizes a single DNA band (∼4.4 kb) in the wild-type strain and a single DNA band (∼2.7 kb) in the Δ*laeA* mutant. Download FIG S7, PDF file, 0.2 MB. Copyright © 2019 Mead et al. 2019 Mead et al. This content is distributed under the terms of the Creative Commons Attribution 4.0 International license.

## DISCUSSION

A. fumigatus is a major human fungal pathogen, yet its close relative *A. fischeri* is rarely an agent of human disease. A number of traits that contribute to the virulence of A. fumigatus have been characterized, but their distribution and potential role in *A. fischeri-*mediated disease are largely unknown. In this study, we thoroughly characterized *A. fischeri* (strain NRRL 181) and compared it to A. fumigatus (strain CEA10) for multiple disease-relevant biological and chemical differences. Our data show that *A. fischeri* can grow in a mammalian host but is much less fit and causes a disease progression quite different than that observed during A. fumigatus infections (Fig. 1 and 2). Future studies will be needed to determine if these differences between *A. fischeri* NRRL 181 and A. fumigatus CEA10 are conserved across a range of *A. fischeri* and A. fumigatus isolates.

Further investigations revealed that secondary metabolic genes are much less conserved than genes in the rest of the genome (see Fig. S3 in the supplemental material), and a chemical analysis of *A. fischeri* resulted in the identification of both previously identified and new compounds (Fig. 5). While the BGCs producing secondary metabolites in *A. fischeri* and A. fumigatus appear to be quite different, our data suggest that a master regulator of secondary metabolism in A. fumigatus (*laeA*) possesses a similar role in *A. fischeri* (Fig. 5C). This is consistent with previous observations that the global regulation of secondary metabolism is conserved but that the underlying BGCs are not, suggesting that the regulatory circuit involved in *Aspergillus* secondary metabolism has undergone extensive regulatory rewiring (41). Our analyses are the first to infer the occurrence of regulatory circuit rewiring in *A. fischeri* and are among the first gene knockout studies of this organism (70).

In order to cause disease, a microbe must be able to respond to the set of diverse and stressful environments presented by its host. Based on our data, *A. fischeri* strain NRRL 181 is unable to respond to many of these stresses as well as A. fumigatus CEA10 (Fig. 2 and 3). We hypothesize that this inability to thrive under stress contributes to the various disease progressions observed during our animal model experiments (Fig. 1). Some or all of the genetic determinants responsible for this discrepancy in stress response and virulence could reside in the ∼1,200 A. fumigatus-specific genes we identified (Fig. S3); alternatively, some of the ∼1,700 *A. fischeri*-specific genes we identified may inadvertently facilitate control of *A. fischeri* in a mammalian host. An additional explanation for the discrepancy in stress response is that A. fumigatus has evolved more efficient regulatory circuits than *A. fischeri* that allow it to cope with more stringent conditions that are observed in the host.

Even though more than 10% of the genes in each species lack an ortholog in the other species, only ∼2% (1/49) of previously identified genetic determinants of virulence in A. fumigatus are not conserved in *A. fischeri* (see Table S1 in the Figshare document at https://doi.org/10.6084/m9.figshare.7149167). This result and our observation that many of the pathways of secondary metabolism are quite different between *A. fischeri* and A. fumigatus support a multifactorial model of A. fumigatus virulence (71–73) and suggest a need to investigate virulence on multiple levels of biological complexity. In order to cause disease in a host, A. fumigatus (and other species closely related to it) must adhere and germinate in the lung (73), survive inherently stressful conditions presented by host environments (e.g., severe lack of metals and oxygen) (5, 74, 75), and modulate or endure actions of the host immune system (76). Given the diversity of these activities, it is unlikely that single genes or pathways will be responsible for the totality of A. fumigatus-derived disease, even though many genes in the genome have not yet been characterized for their role in pathogenicity. We hypothesize that multiple pathways (including those involved in secondary metabolism) have changed during the evolution of *A. fischeri* and A. fumigatus, resulting in their differing ability to cause disease.

As mentioned, an important caveat to our experiments is that we analyzed only a single, representative strain from each species, and further studies are needed to determine how typical these observed trait and genomic differences are across multiple strains from each species. Several recent studies have identified a wide variety of differences between A. fumigatus strains, which have in turn been shown to contribute to physiological differences, including but not limited to secondary metabolism and virulence (30, 72, 73). While the genome of only one isolate of *A. fischeri* has so far been sequenced (16) and the organism has been reported to cause human disease only a few times (21–24), it would be of great interest to compare patient-derived and environment-derived isolates at the genomic, phenotypic, and chemical levels. Although it appears that clinical and environmental isolates do not stem from separate lineages in A. fumigatus (77), whether this is also the case for largely nonpathogenic species, such as *A. fischeri*, or for rarely isolated pathogenic species, such as A. lentulus or *A. udagawae*, remains largely unknown.

Our mouse infection studies revealed that *A. fischeri* strain NRRL 181 was less virulent than A. fumigatus strain CEA10 in both leukopenic and steroid-induced immune suppression models of disease. The difference in disease outcome in the steroid model closely matches what was observed in comparisons of two A. fumigatus strains, CEA10 and Af293 (30). It is intriguing that the difference between pathogenic (A. fumigatus CEA10) and nonpathogenic (*A. fischeri* NRRL 181) species in disease outcomes in this model is similar to that between two clinically derived pathogenic strains (A. fumigatus CEA10 and Af293). Interestingly, Kowalski et al. (30) did not observe any statistically significant difference in disease progression between CEA10 and Af293 in the leukopenic model, whereas we found that *A. fischeri* is less virulent in this second murine model of disease (Fig. 1). These strain-, species-, and infection model-specific differences in disease outcomes are consistent with our hypothesis that A. fumigatus virulence is a complex genetic trait and highlight the need for further studies using wider sets of strains, species, and virulence-related traits.

A. fumigatus and *A. fischeri* are members of *Aspergillus* section *Fumigati*, a clade that includes multiple closely related species, some of which are pathogens (e.g., A. fumigatus, A. lentulus, and *A. udagawae*) and some of which are considered nonpathogens (e.g., *A. fischeri*, A. aureolus, and A. turcosus) (1, 44, 78, 79). The ability to cause disease in humans appears to have either arisen or been lost (or both) multiple times independently during the evolution of this lineage, as pathogenic species are spread throughout the phylogeny (17, 80). A broader, phylogenetically informed comparison of pathogenic and nonpathogenic species in section *Fumigati* would provide far greater resolution in identifying (or dismissing) factors and pathways that may contribute to or prevent the ability of these organisms to cause disease. Also, leveraging the diversity of section *Fumigati* would give researchers a better understanding of the nature and evolution of human fungal pathogenesis as the appreciation for the health burden caused by fungi increases (81).

## MATERIALS AND METHODS

### Strains and growth media.

*A. fischeri* strain NRRL 181 was acquired from the ARS Culture Collection (NRRL). A. fumigatus strain CEA10 (CBS 144.89) was obtained from the Westerdijk Fungal Biodiversity Institute (CBS). All strains were grown on glucose minimal medium (GMM) from conidial glycerol stocks stored at −80°C. All strains were grown in the presence of white light at 37°C. Conidia were collected in 0.01% Tween 80 and enumerated with a hemocytometer.

### Murine virulence studies.

For the chemotherapeutic (leukopenic) murine model, outbred CD-1 female mice (Charles River Laboratories, Raleigh, NC, USA), 6 to 8 weeks old, were immunosuppressed with intraperitoneal (i.p.) injections of 150 mg/kg (of body weight) cyclophosphamide (Baxter Healthcare Corporation, Deerfield, IL, USA) 48 h before and 72 h after fungal inoculation, along with subcutaneous (s.c.) injections of 40 mg/kg triamcinolone acetonide (Kenalog-10; Bristol-Myers Squibb, Princeton, NJ, USA) 24 h before and 6 days after fungal inoculation. For the murine triamcinolone model, outbred CD-1 female mice, 6 to 8 weeks old, were treated with 40 mg/kg triamcinolone acetonide by s.c. injection 24 h prior to fungal inoculation.

Unless otherwise noted, conidial suspensions of 2 × 10^6^ conidia were prepared in 40 μl sterile PBS and administered to mice intranasally while under isoflurane anesthesia. Mock mice were given 40 μl PBS. Mice were monitored three times a day for signs of disease for 14 or 18 days postinoculation. Survival was plotted on Kaplan-Meier curves, and statistical significance between curves was determined using Mantel-Cox log rank and Gehan-Breslow-Wilcoxon tests. Mice were housed in autoclaved cages at 4 mice per cage with HEPA-filtered air and autoclaved food and water available *ad libitum*.

### Galleria mellonella virulence studies.

G. mellonella larvae were obtained by breeding adult moths (82). G. mellonella larvae of a similar size were selected (approximately 275 to 330 mg) and kept without food in glass containers (petri dishes) at 37°C in darkness for 24 h prior to use. A. fumigatus and *A. fischeri* conidia were obtained by growth on YAG medium for 2 days. The conidia were harvested in PBS and filtered through Miracloth (Calbiochem). The concentration of conidia was estimated by using a hemocytometer, and conidia were resuspended at a concentration of 2.0 × 10^8^ conidia/ml. The viability of the conidia was determined by incubation on YAG medium at 37°C for 48 h. Inocula (5 µl) of conidia from both strains were used to investigate the virulence of A. fumigatus and *A. fischeri* against G. mellonella. Ten G. mellonella larvae in the final (sixth) instar larval stage of development were used per condition in all assays. The control group was the larvae inoculated with 5 μl of PBS to observe the killing due to physical trauma. The inoculation was performed by using a Hamilton syringe (7000.5KH) and injecting 5 μl into the hemocoel of each larva via the last left proleg. Afterward, the larvae were incubated in a glass container (petri dishes) at 37°C in the dark. Larval killing was scored daily. Larvae were considered dead by presenting the absence of movement in response to touch.

### Histopathology.

Outbred CD-1 mice, 6 to 8 weeks old, were immunosuppressed and intranasally inoculated with 2 × 10^6^ conidia as described above for the chemotherapeutic and corticosteroid murine models. Mice were sacrificed 72 h postinoculation. Lungs were perfused with 10% buffered formalin phosphate before removal and then stored in 10% buffered formalin phosphate until embedding. Paraffin-embedded sections were stained with hematoxylin and eosin (H&E) and Gomori methenamine silver (GMS). Slides were analyzed microscopically with a Zeiss Axioplan 2 imaging microscope (Carl Zeiss Microimaging, Inc., Thornwood, NY, USA) fitted with a QImaging Retiga-SRV Fast 1394 RGB camera. Analysis was performed in Phylum Live 4 imaging software.

### Ethics statement.

We carried out our mouse studies in strict accordance with the recommendations in the *Guide for the Care and Use of Laboratory Animals* of the National Research Council (83). The mouse experimental protocol was approved by the Institutional Animal Care and Use Committee (IACUC) at Dartmouth College (Federal-wide assurance number A3259-01).

### Growth assays.

Radial growth was quantified by point inoculation of 1 × 10^3^ conidia in 2 μl on indicated media; plates were incubated at 37°C in normoxia (∼21% O_2_, 5% CO_2_) or hypoxia (0.2% O_2_, 5% CO_2_). Colony diameter was measured every 24 h for 4 days and reported as the average from three biological replicates per strain.

For 2-DG experiments, 1 × 10^3^ conidia in 2 μl were spotted on 1% lactate minimal medium with or without 0.1% 2-deoxyglucose (2-DG; Sigma, D8375). Plates were incubated for 3 days at 37°C in normoxia or hypoxia with 5% CO_2_. Percent inhibition was calculated by dividing radial growth on 2-DG plates by the average radial growth of biological triplicates on plates without 2-DG.

Fungal biomass was quantified by measuring the dry weight of fungal tissue from 5 × 10^7^ conidia grown in 100 ml liquid GMM with shaking at 200 rpm for 48 h in normoxia (∼21% O_2_) and hypoxia (0.2% O_2_, 5% CO_2_). Liquid biomass is reported as the average from three biological replicates per strain. Hypoxic conditions were maintained using an InvivO_2_ 400 hypoxia workstation (Ruskinn Technology Limited, Bridgend, United Kingdom) with a gas regulator and 94.8% N_2_.

Liquid growth curves were performed with conidia adjusted to 2 × 10^4^ conidia in 20 μl 0.01% Tween 80 in 96-well dishes, and then 180 μl of medium (GMM or lung homogenate) was added to each well. Plates were incubated at 37°C for 7 h, and then *A*_405_ measurements were taken every 10 min for the first 16 h of growth with continued incubation at 37°C. Lung homogenate medium was prepared as follows: lungs were harvested from healthy CD-1 female mice (20 to 24 g) and homogenized through a 100-μm cell strainer in 2 ml PBS/lung. Homogenate was diluted 1:4 in sterile PBS, spun down to remove cells, and then filter sterilized through 22-μm PVDF filters.

### Cell wall and oxidative stresses.

Congo red (0.5 mg/ml), menadione (20 µM), or calcofluor white (CFW; 25 µg/ml) was added to GMM plates. Conidia at 1 × 10^3^ (calcofluor white and menadione) or 1 × 10^5^ (Congo red) were point inoculated, and plates were incubated for 96 h at 37°C with 5% CO_2_.

### Orthology determination and analyses.

Genomes for A. fumigatus CEA10 and *A. fischeri* NRRL 181 were downloaded from NCBI (accession numbers GCA_000150145.1 and GCF_000149645.1, respectively). To identify putative orthologous genes between *A. fischeri* and A. fumigatus, a reciprocal best BLAST hit (RBBH) approach was used. We performed a BLAST search on the proteome of *A. fischeri* to A. fumigatus and vice versa using an E value cutoff of 10^−3^ and then filtered for RBBHs according to bitscore (84). A pair of genes from each species was considered orthologous if their best BLAST hit was to each other. Species-specific and orthologous protein sets were visualized using version 3.0.0 of eulerAPE (85).

### Biosynthetic gene cluster (BGC) prediction and analyses.

Version 4.2.0 of antiSMASH (40) was used with its default settings to identify BGCs. Orthologous cluster genes were identified using our RBBH results and visualized using version 0.69 of Circos (86). Chromosomes were identified for *A. fischeri* NRRL1 and A. fumigatus CEA10 using NUCMER (87) and chromosomal sequences from A. fumigatus strain AF293 from NCBI (accession number GCA_000002655.1). Syntenic clusters were visualized using Easyfig version 2.2.2 (88).

### Secondary metabolite extraction and identification.

Secondary metabolites were extracted from *A. fischeri* using techniques well established in the natural product literature (89, 90). This was done by adding a 1:1 mixture of CHCl_3_-CH_3_OH and leaving the mixture to shake overnight. The resulting slurry was partitioned twice, first with a 4:1:5 CHCl_3_-CH_3_OH-H_2_O solution, with the organic layer drawn off and evaporated to dryness *in vacuo*, and second, reconstituting 1:1:2 CH_3_CN-CH_3_OH-hexanes, where the organic layer was drawn off and evaporated to dryness. The extract then underwent chromatographic separation (flash chromatography and HPLC) using varied gradient systems. The full structural characterization of the new secondary metabolites is provided in a Figshare document (https://doi.org/10.6084/m9.figshare.7149167).

### Construction of the *A. fischeri* Δ*laeA* mutant.

The gene replacement cassettes were constructed by *in vivo* recombination in Saccharomycescerevisiae as previously described (91, 92). Approximately 2.0 kb from the 5′-UTR and 3′-UTR flanking regions of the targeted ORF were selected for primer design. The primers pRS NF010750 5′fw (5′-GTAACGCCAGGGTTTTCCCAGTCACGACGCAGTCTAACGCTGGGCCCTTCC-3′) and pRS NF010750 3′rv (5′-GCGGTTAACAATTTCTCTCTGGAAACAGCTACGGCGTTTGACGGCACAC-3′) contained a short homologous sequence to the multicloning site (MCS) of the plasmid pRS426. Both the 5′- and 3′-UTR fragments were PCR amplified from *A. fischeri* genomic DNA (gDNA). The *prtA* gene, conferring resistance to pyrithiamine, which was placed within the cassette as a dominant marker, was amplified from the pPRT1 plasmid by using the primers prtA NF010750 5′rv (5′-GTAATCAATTGCCCGTCTGTCAGATCCAGGTCGAGGAGGTCCAATCGG-3′) and prtA NF010750 3′fw (5′-CGGCTCATCGTCACCCCATGATAGCCGAGATCAATCTTGCATCC-3′). The deletion cassette was generated by transforming each fragment along with the plasmid pRS426, cut with BamHI/EcoRI, into the S. cerevisiae strain SC94721 using the lithium acetate method (93). The DNA from the transformants was extracted by the method described by Goldman et al. (94). The cassette was PCR amplified from these plasmids utilizing TaKaRa *Ex Taq* DNA polymerase (Clontech TaKaRa Bio) and used for *A. fischeri* transformation according to the protocol described by Malavazi and Goldman (92). Southern blotting and PCR analyses were used to demonstrate that the cassette had integrated homologously at the targeted *A*. *fischeri* locus. Genomic DNA from *A*. *fischeri* was extracted by grinding frozen mycelia in liquid nitrogen, and then gDNA was extracted as previously described (92). Standard techniques for manipulation of DNA were carried out as described previously (95). For Southern blot analysis, restricted chromosomal DNA fragments were separated on a 1% agarose gel and blotted onto Hybond N+ nylon membranes (GE Healthcare). Probes were labeled using [α-^32^P]dCTP using the Random Primers DNA labeling system (Life Technologies). Labeled membranes were exposed to X-ray films, which were scanned for image processing. Southern blotting and PCR schemes are shown in Fig. S7 in the supplemental material.
