# Supplementary figures and images for: Characterizing the Pathogenic, Genomic, and Chemical Traits of Aspergillus fischeri, a Close Relative of the Major Human Fungal Pathogen Aspergillus fumigatus
Source: mSphere. 2019 Feb 20;4(1):e00018-19. doi: 10.1128/mSphere.00018-19 (PMC6382966; doi:10.1128/mSphere.00018-19)

Figure S1

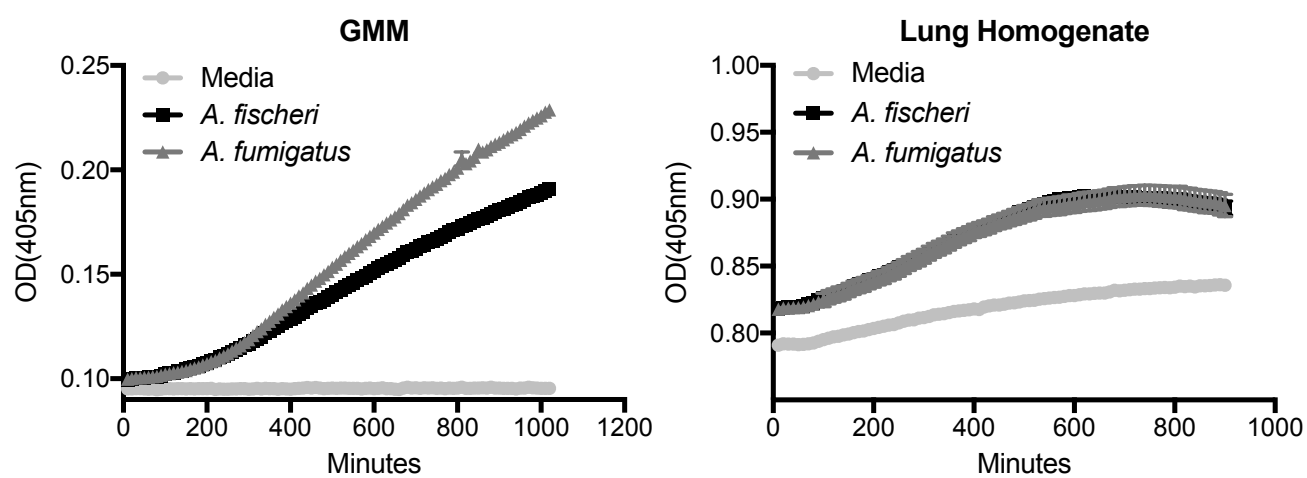

Supplement: FIG S1 [file mSphere.00018-19-sf001.pdf]

# Figure S2

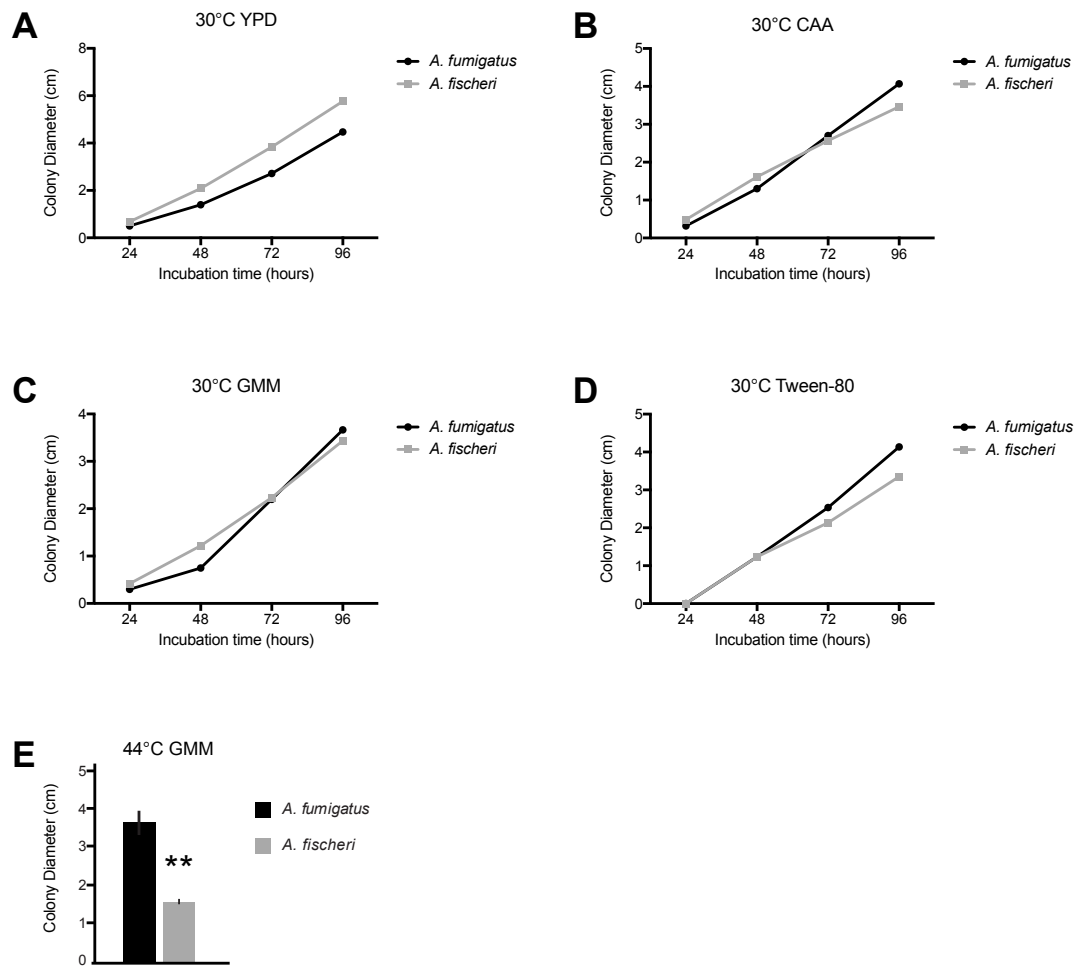

Supplement: FIG S2 [file mSphere.00018-19-sf002.pdf]

Figure S3

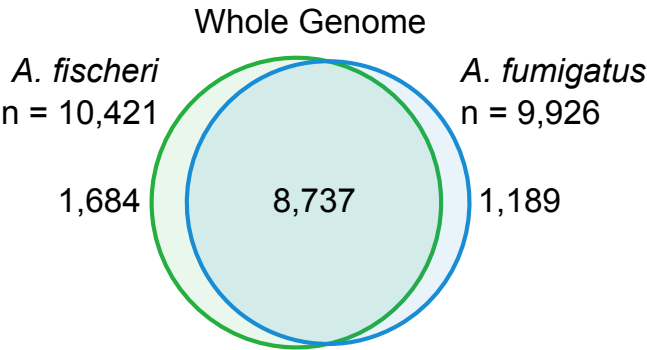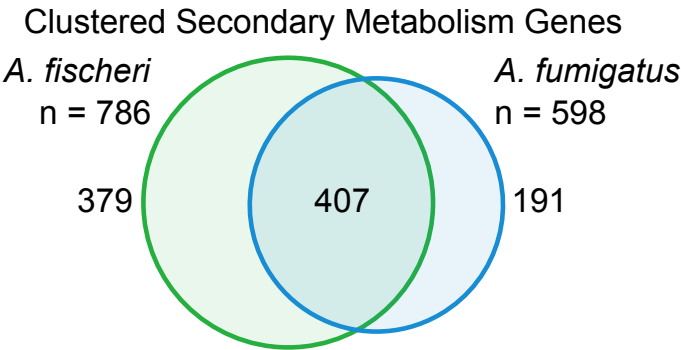

Supplement: FIG S3 [file mSphere.00018-19-sf003.pdf]

# Figure S4

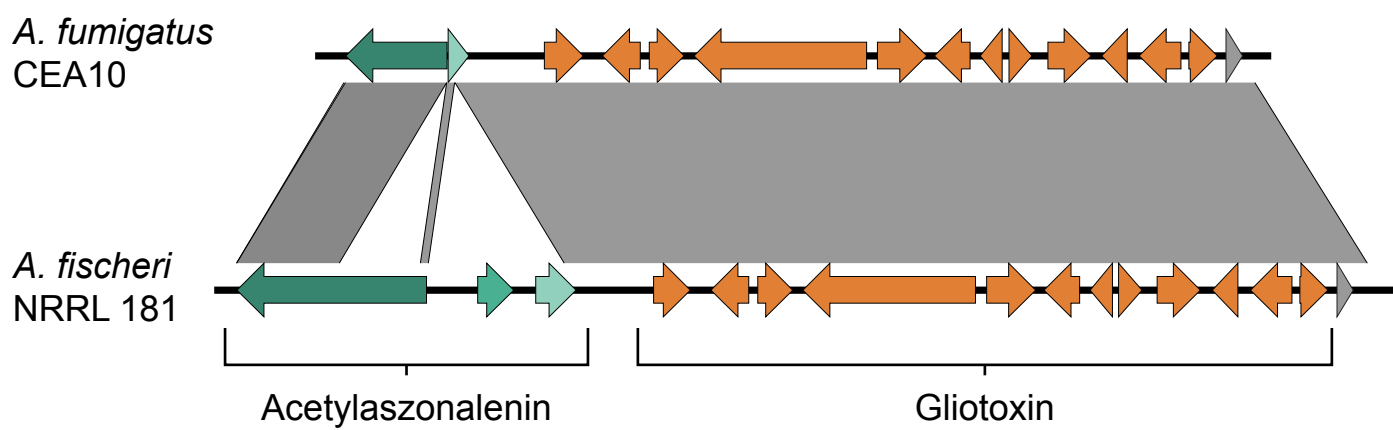

Supplement: FIG S4 [file mSphere.00018-19-sf004.pdf]

# Figure S5

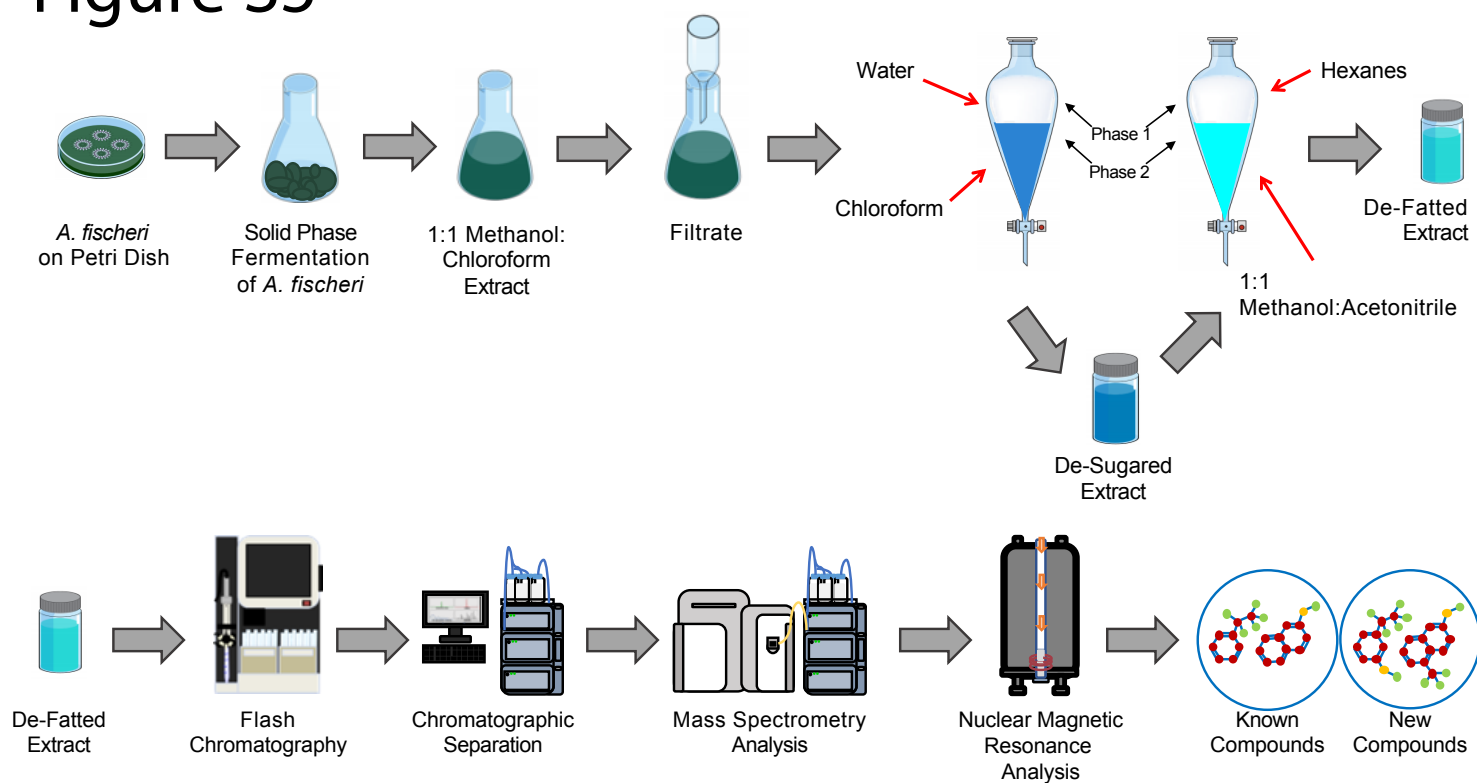

Supplement: FIG S5 [file mSphere.00018-19-sf005.pdf]

Figure S6

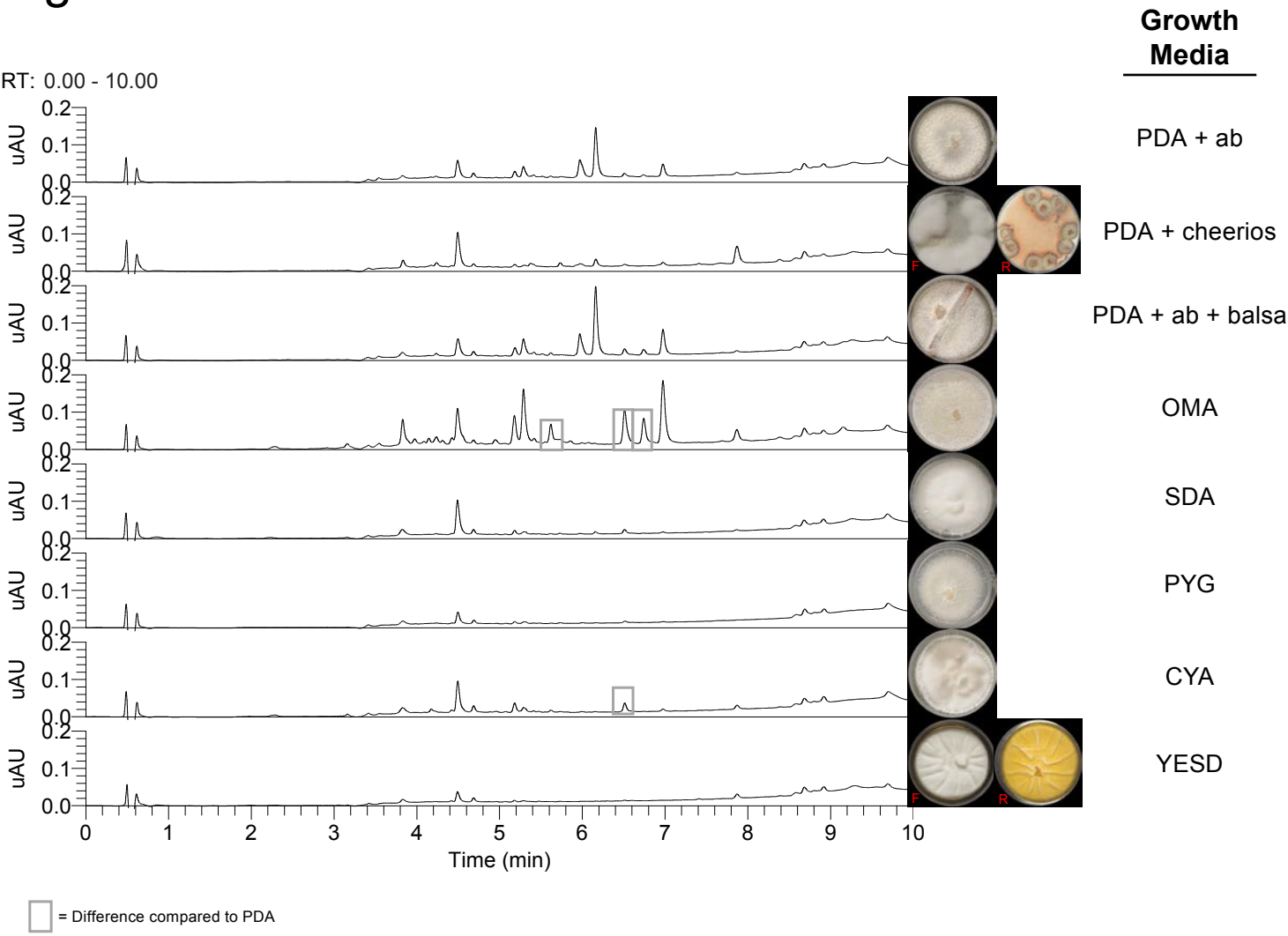

Supplement: FIG S6 [file mSphere.00018-19-sf006.pdf]

Figure S7

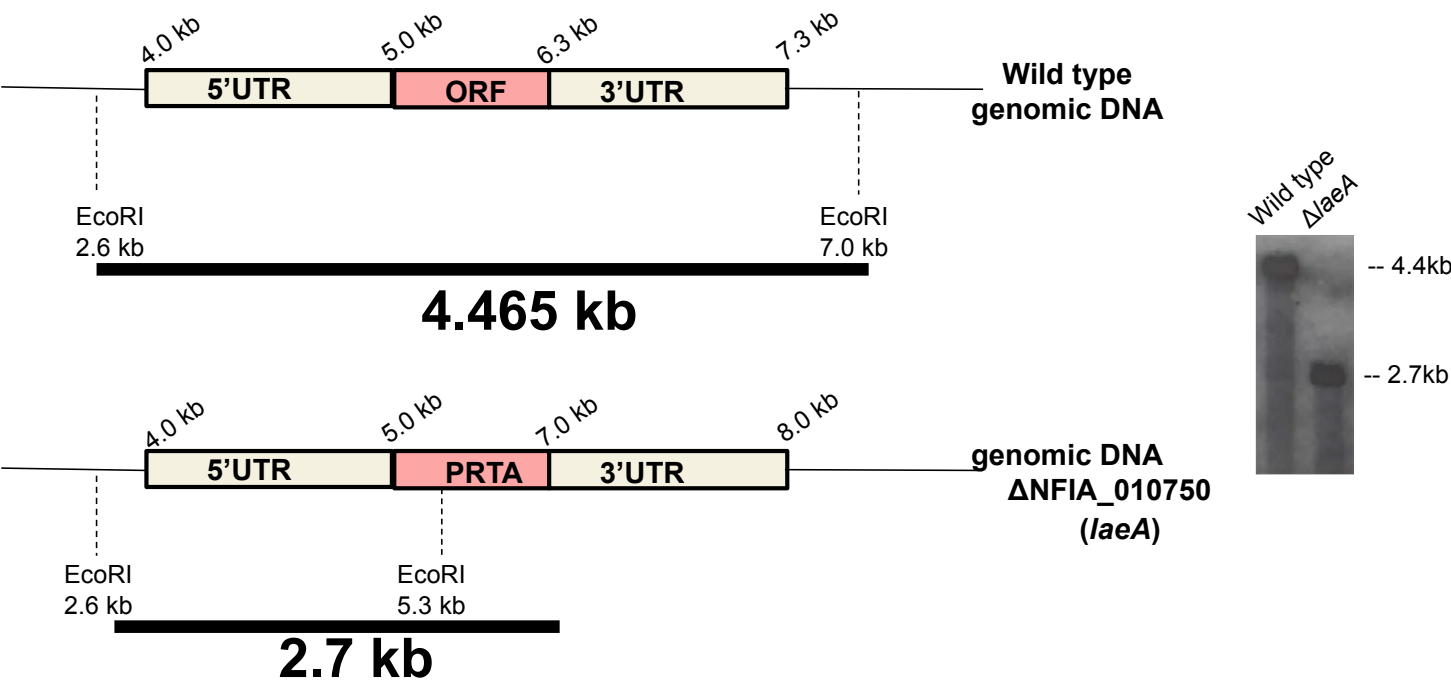

Supplement: FIG S7 [file mSphere.00018-19-sf007.pdf]
